# GRPR-Antagonists Carrying DOTAGA-Chelator via Positively Charged Linkers: Perspectives for Prostate Cancer Theranostics

**DOI:** 10.3390/pharmaceutics16040513

**Published:** 2024-04-08

**Authors:** Karim Obeid, Panagiotis Kanellopoulos, Ayman Abouzayed, Adam Mattsson, Vladimir Tolmachev, Berthold A. Nock, Theodosia Maina, Anna Orlova

**Affiliations:** 1 Department of Medicinal Chemistry, Uppsala University, 751 83 Uppsala, Sweden; karim.obeid.1838@student.uu.se (K.O.); panagiotis.kanellopoulos@ilk.uu.se (P.K.); ayman.abouzayed@ilk.uu.se (A.A.); adam.mattsson.4918@student.uu.se (A.M.); 2 Molecular Radiopharmacy, INRaSTES, NCSR “Demokritos”, 15341 Athens, Greece; nock_berthold.a@hotmail.com (B.A.N.); maina_thea@hotmail.com (T.M.); 3 Department of Immunology, Genetics and Pathology, Uppsala University, 751 83 Uppsala, Sweden; vladimir.tolmachev@igp.uu.se; 4 Science for Life Laboratory, Uppsala University, 752 37 Uppsala, Sweden

**Keywords:** prostate cancer, GRPR, GRPR-antagonist, radiotheranostics, PC-3 cell/tumor, neprilysin, positively charged linker

## Abstract

Gastrin-releasing peptide receptor (GRPR)-antagonists have served as motifs in the development of theranostic radioligands for prostate cancer. Our efforts have been focused on the development of radiolabeled RM26 (H-DPhe^6^–Gln^7^–Trp^8^–Ala^9^–Val^10^–Gly^11^–His^12^–Sta^13^–Leu^14^–NH_2_) analogs, such as [^111^In]In-DOTAGA-PEG2-RM26. We recently showed that its Gly^11^/Sar^11^-substituted version, [^111^In]In-AU-RM26-M1, resisted degradation by neprilysin (NEP) while in circulation and achieved higher tumor uptake in mice. We herein introduce the following three new AU-RM26-M1 mimics labeled with In-111, with basic residues in the linker: (i) AU-RM26-M2 (PEG2-Pip), (ii) AU-RM26-M3 (PEG2-Arg), and (iii) AU-RM26-M4 (Arg-Arg-Pip). These analogs were compared in PC-3 cells and animal models vs. AU-RM26-M1 (reference). The new analogs showed high affinity and specificity for the GRPR, exhibiting an uptake and distribution pattern in PC-3 cells typical for a radiolabeled GRPR-antagonist. They showed high stability in peripheral mice blood, except for [^111^In]In-AU-RM26-M3. AU-RM26-M4 achieved the highest tumor uptake and promising background clearance, followed by [^111^In]In-RM26-M2, showing lower background levels. These findings were confirmed for [^111^In]In-AU-RM26-M2 and [^111^In]In-AU-RM26-M4 by micro-SPECT/CT at 4 and 24 h post-injection. Hence, the type of positively charged residues in the linker of AU-RM26-M1 mimics strongly influenced biological behavior. The analogs with Pip next to DPhe^6^ demonstrated the best overall characteristics and warrant further investigation.

## 1. Introduction

Prostate cancer affects one in seven men, representing the second most common malignancy in men worldwide [1,2]. Early diagnosis is essential for a good prognosis, but most diagnostic tools today present a number of shortcomings. Compromised diagnostic accuracy translates to sub-optimal therapy planning and outcome. However, recently, promising new opportunities have become available to prostate cancer patients via the so-called “radiotheranostic” concept in nuclear medicine [3,4]. Accordingly, diagnostic imaging is performed first with a diagnostic radiotracer suitable for SPECT (single photon emission computed tomography) or PET (positron emission tomography) to select patients eligible for radionuclide therapy. Imaging provides valuable information on disease stage and spread, the expression of the biomolecular target on tumor lesions, and allows for dosimetric calculations. Based on these findings, radionuclide therapy is carefully planned in a patient-tailored fashion with the respective therapeutic radiopharmaceutical delivering radiotoxic payloads specifically to tumor sites, sparing healthy tissues. This concept has been clinically established in prostate cancer with the advent of radiolabeled prostate-specific membrane antigen (PSMA)-inhibitors [5]. PSMA is highly expressed in prostate cancer, but still, its expression varies across patients and disease states, leaving space for other targeting options [6,7,8,9].

In this regard, the gastrin-releasing peptide receptor (GRPR), overexpressed in various human cancers, including prostate cancer, represents a valid biomolecular target in nuclear medicine [10,11,12,13,14,15,16]. Most interestingly, GRPR is highly expressed in early-stage prostate cancer with advantageously no expression in benign prostatic hyperplasia [15,16,17]. On the other hand, GRPR expression patterns become less consistent in metastatic advanced stages of the disease [9]. Numerous theranostic radiopharmaceuticals with promising preclinical results for use in the management of prostate cancer have been developed over the years based on GRPR-antagonists [7,18,19]. Antagonists are the preferred option for injection into patients because they do not activate the GRPR upon binding and hence do not elicit acute adverse effects. Furthermore, radiolabeled GRPR-antagonists have shown superior pharmacokinetic profiles compared with agonists in animal models and humans [18].

Most GRPR-antagonist motifs used in radiopharmaceutical design are analogs of the amphibian tetradecapeptide bombesin (BBN, Pyr-Gln-Arg-Leu-Gly-Asn-Gln-Trp-Ala-Val-Gly-His-Leu-Met-NH_2_) [20] and especially the C-terminal nonapeptide [H-DPhe^6^]BBN(6–14) fragment, retaining full capability to interact with the GRPR [7]. Suitable structural interventions in the C-terminal Leu^13^-Met^14^-NH_2_ dipeptide have led to potent GRPR-antagonists [21]. Thus, replacement of Leu^13^-Met^14^-NH_2_ by Sta^13^-Leu^14^-NH_2_ (Sta, 4-amino-3-hydroxy-6-methylheptanoic acid) yields the potent GRPR-antagonist JMV594 (or RM26) [22], which has been broadly used as a motif in radioligand design [7,23]. Of special interest is RM2, whereby the chelator DOTA (1,4,7,10-tetraazacyclododecane-1,4,7,10-tetraacetic acid) is covalently attached at the N-terminal DPhe^6^ of RM26 via a Pip (Pip, 4-amino-1-carboxymethyl-piperidine) linker [23], allowing for labeling with theranostic trivalent radiometals (e.g., Ga-68, In-111, or Lu-177) [24].

We have likewise developed a number of RM2-related analogs as radiopharmaceutical candidates for prostate cancer theranostics, carrying different types of radiometal chelators via diverse linkers [7,25]. To implement targeted therapy, we have introduced the DOTAGA (1,4,7,10-tetrakis(carboxymethyl)-1,4,7,10-tetraazacyclo-dodecane glutaric acid) chelator in several analogs, known to form stable complexes with a number of therapeutic radiometals, including beta (Lu-177, Y-90) and alpha emitters (Ac-211, Bi-213) [24]. Aiming toward higher metabolic stability, we proposed the Gly^11^ to Sar^11^ (sarcosine, N-methyl-glycine) substitution in the new analogs, previously shown to enhance resistance to the fast proteolytic action of neprilysin (NEP) in blood circulation [18]. In fact, we recently demonstrated the improved metabolic stability and enhanced tumor uptake of [^111^In]In-DOTAGA-PEG2-[Sar^11^]RM26 ([^111^In]In-AU-RM26-M1) in a head-to-head comparison study with unmodified [^111^In]In-DOTAGA-PEG2-RM26 [26].

**Figure 1:**
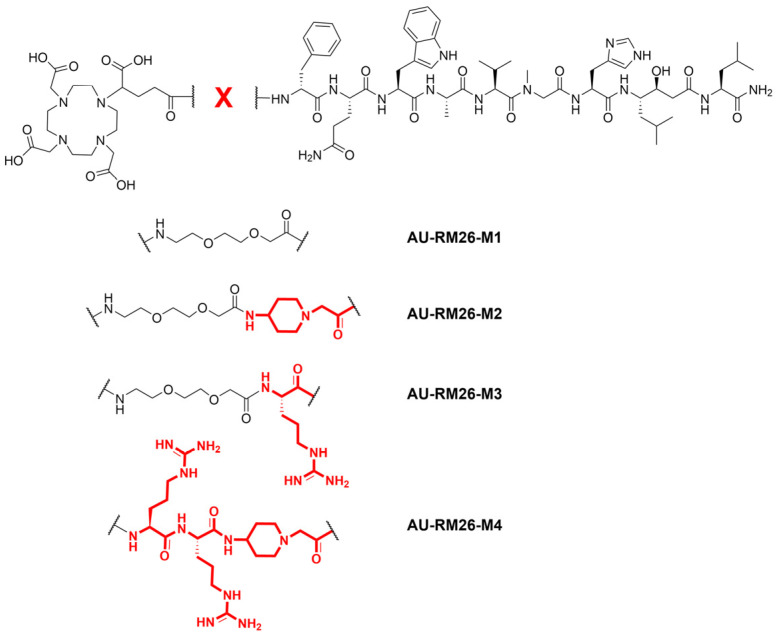
Chemical structures of AU-RM26-M1 reference (DOTAGA-PEG2-[Sar^11^]RM26), AU-RM26-M2 (DOTAGA-PEG2-Pip-[Sar^11^]RM26), AU-RM26-M3 (DOTAGA-PEG2-Arg-[Sar^11^]RM26) and AU-RM26-M4 (DOTAGA-Arg-Arg-Pip-[Sar^11^]RM26). Structural differences in linkers are marked in red.

Aiming at further improvements of the pharmacokinetic profile of [^111^In]In-AU-RM26-M1, we next directed our efforts toward prolonging tumor retention and increasing clearance from healthy tissues, especially from excretory organs (like the kidneys), thereby maximizing the therapeutic index. For this purpose, we introduced three new [^111^In]In-AU-RM26-M1 analogs, carrying different types and numbers of basic residues in the linker. Previous studies have indicated that positively charged residues next to the receptor-recognizing peptide moiety may increase receptor affinity, cell binding and tumor uptake of radiolabeled BBN-like peptides [19,27,28]. On the other hand, basic residues carrying pendant primary amines (e.g., Lys) were shown to unfavorably increase renal accumulation [7,19,29]. Taking all the above into consideration, we herein introduce the following analogs: (i) AU-RM26-M2 with the PEG2-Pip linker, (ii) AU-RM26-M3 with a PEG2-Arg linker, and (iii) AU-RM26-M4 with an Arg-Arg-Pip linker (Figure 1). Thus, the type, number and position of the positively charged residues have been varied in these analogs, excluding residues with lateral primary amines. The biological profile of the new bioconjugates after labeling with In-111 was evaluated in direct comparison with [^111^In]In-AU-RM26-M1 in GRPR-expressing prostate adenocarcinoma PC-3 cells [30] and mice models, and their potential discussed as radiotherapeutic candidates in prostate cancer after labeling with particle emitting radiometals, like Lu-177.

## 2. Materials and Methods

### 2.1. Peptides and Reagents

The peptides AU-RM26-M1, AU-RM26-M2, AU-RM26-M3, and AU-RM26-M4 were synthesized by Pepmic Co., Ltd. (Suzhou, China). The GRPR-positive prostate cancer cell line (PC-3) was purchased from American Type Culture Collection (ATCC) (Manassas, VA, USA) and maintained in Roswell Park Memorial Institute (RPMI) 1640 media supplemented with 10% of fetal bovine serum and 1% of penicillin–streptomycin (100 IU/mL penicillin, 100 μg/mL streptomycin) in a humidified atmosphere with 5% CO_2_ at 37 °C in a Sanyo MCO-19AIC incubator (SANYO Electric Co., Ltd., Osaka City, Osaka, Japan). Cellular detachment was performed using a 0.25% trypsin-EDTA solution. Media supplements and trypsin were purchased from Biochrom AG (Berlin, Germany). Indium-111 was obtained as [^111^In]InCl_3_ from Curium Pharma (Stockholm, Sweden). The in vitro binding specificity assay was performed on a 35 mm 6-well plate purchased from VWR International (Radnor, PA, USA). The affinity measurements were conducted on 89 mm Petri dishes (Nunclon Delta Surface, ThermoFisher Scientific, Roskilde, Denmark). Cellular internalization was performed on 35 mm cell culture dishes provided by Corning Inc. (Corning, NY, USA). The radioactivity content of all assay samples was measured using the Wizard2TM gamma counter (PerkinElmer, Waltham, MA, USA).

### 2.2. Radiolabeling and Radiochemical Studies

The labeling of all compounds was performed successfully following the same protocol. The peptides were radiolabeled with In-111 by adding [^111^In]InCl_3_ (3.4–56.8 MBq, 6.8–160 μL) to 2 μL of peptide (1 mM) in 60–80 μL of ammonium acetate buffer (0.2 M, pH 5.5) and 10 μL of ascorbic acid (0.1 M), which was used as an antioxidant. This mixture was incubated at 85 °C for 30 min.

The reaction mixture was analyzed by high-performance liquid chromatography (HPLC) on a system comprising a LaPrep Sigma HPLC LP1100 pump (Hitachi High-Tech Corporation, Hitachinaka, Ibaraki, Japan) equipped with a 40D LWL UV detector with a 4 μL flow cell (Knauer, Berlin, Germany), a flow scan radioactivity detector (Bioscan) with an FC-3300 NaI/PMT radioactivity probe (Eckert & Ziegler, Berlin, Germany) and a manual simple injector 7725i, by Rheodyne, fitted with a 20 μL loop (IDEX Health & Science, LLC, Rohnert Park, CA, USA). Data analysis and instrument monitoring were performed using Open Lab EZChrome Elite 3.2.0 software (Agilent, Santa Clara, CA, USA). A Luna C18 column (5 μm, 100 Å, 150 × 4.6 mm from Phenomenex, Værløse, Denmark) was eluted with 0.1% *v*/*v* aqueous trifluoroacetic acid (TFA) (A) and 0.1% *v*/*v* TFA in acetonitrile (MeCN) (B), adopting the following elution gradient: 0–15 min from 95% A/5% B to 30% A/70% B, 15–17 min 30% A/70% B to 5% A/95% B, 17–19 min at 5% A/95% B, and 19–20 min final reconditioning to 95% A/5% B (system 1).

The radiochemical yield was analyzed with instant thin layer chromatography (iTLC) strips (Agilent Technologies, Santa Clara, CA, USA), using 0.2 M citric acid buffer as a mobile phase (R_f_ = 0 for the radiolabeled peptide and R_f_ = 1 for free indium). The iTLC results were analyzed using Cyclone^®^ Plus Phosphorimager (PerkinElmer, Hägersten, Sweden). The radiochemical stability was determined by incubating the radiolabeled compounds with phosphate-buffered saline (PBS) or a 1000-fold molar excess of ethylenediaminetetraacetic acid (EDTA) for 1 h at room temperature. The percentage of indium release was assessed by iTLC using 0.2 M citric acid as mobile phase. Stability tests were performed in triplicate for each compound.

### 2.3. In Vitro Studies

#### 2.3.1. In Vitro Binding Specificity Assay

The in vitro binding assay was performed on PC-3 cells seeded in 6-well plates (1.15 × 10^6^ cells/well). Media containing a GRPR-blocking agent (NOTA-PEG2-RM26, 1 μM) [26] were added to half of the wells. The plates were incubated at room temperature for 10 min and the medium containing the radiotracer under investigation was added to all wells in a final concentration of 1 nM. The plates were incubated for 1 h and then treated with 0.25% trypsin-EDTA to detach cells. The cells were collected and their activity was measured using the gamma counter.

#### 2.3.2. Affinity Measurements

The affinities of [^111^In]In-AU-RM26-M2, [^111^In]In-AU-RM26-M3 and [^111^In]In-AU-RM26-M4 for the human GRPR were measured on PC-3 cells seeded on Petri dishes (3 × 10^6^ cells/dish) at room temperature in real-time using a LigandTracer Yellow Instruments (Ridgeview Instruments AB, Uppsala, Sweden). The association curves were recorded at 1 nM and 3 nM of the radiolabeled peptides for 300 min. After reaching the plateau, the medium containing the radioligand was replaced with fresh medium and the dissociation curve was measured for about 16 h. The obtained sensorgrams were analyzed using TracerDrawer (Ridgeview Instruments AB, Uppsala, Sweden) and the dissociation constants (K_D_) were calculated.

#### 2.3.3. Cellular Internalization

For the cellular internalization assay, PC-3 cells (1 × 10^6^ cells/dish) were used. The cells were incubated with 1 mL (1 nM) of solution containing the radiolabeled peptide under evaluation. At the 4 h and 24 h time points, the cells were treated with an acid wash (0.2 M glycine buffer with 0.15 M NaCl and 4 M urea, pH 2) for 5 min on ice to remove the membrane-bound peptide. After collection of acid fractions, the cells were treated with 1 M NaOH for at least 30 min and the cell debris were scraped and collected. The activity content in samples was measured using the gamma counter.

### 2.4. In Vivo Studies

All in vivo studies were performed following European guidelines on laboratory animal protection. The in vivo stability experiments were carried out in healthy male Swiss albino mice; the study protocol was approved by the Department of Agriculture and Veterinary Service of the Prefecture of Athens (protocol number #440448, 01-06-2021). The biodistribution, in vivo targeting specificity assay, and SPECT/CT imaging experiments were carried out on BALB/C nu/nu mice. The Ethics Committee for Animal Research in Uppsala (Sweden) approved the latter in vivo animal studies (permit number 00473/21). The BALB/C nu/nu mice were implanted with GRPR-positive prostate cancer xenografts by subcutaneous injection on the right hind leg of PC-3 cells suspended in PBS (7 × 10^6^ cells/mouse) four weeks before the biodistribution studies.

#### 2.4.1. In Vivo Stability Experiments

Healthy male Swiss albino mice in groups of three (30 ± 5 g, NCSR “Demokritos” Animal House, Athens, Greece) were intravenously (iv) injected with a bolus containing the test radioligand (100 μL, 2 nmol in PBS/EtOH *v*/*v* 9/1; controls). For NEP inhibition, a parallel group of mice received by gavage a slurry of Entresto^®^ (Novartis, Basel, Switzerland) 30 min prior to radioligand injection (individual doses of 12 mg/200 μL dose per animal; Entresto^®^ groups). Entresto^®^ pills (200 mg containing 24 mg/26 mg sacubitril/valsartan; Novartis AG, Basel, Switzerland) purchased from a local pharmacy were ground to a fine powder in a mortar [31,32,33,34]. They were then suspended in tap water to form a slurry and equally distributed in individual portions for oral gavage to mice (12 mg/200 μL per animal) [34]. Mice were euthanized 5 min post-injection (pi) and blood samples were rapidly drawn from the heart in 1.5 mL LoBind Eppendorf tubes (kept at 0 °C) containing EDTA (0.1 mM, 20 μL). Samples were centrifuged at 2000× *g* for 10 min at 4 °C. The plasma was collected and diluted in a 1:1 *v*/*v* ratio with MeCN and the samples were centrifuged postpoagain at 15,000× *g* for 10 min at 4 °C. The supernatant was collected and transferred in a glass vial and concentrated to a final volume of 50–100 μL under mild heating at 50 °C and a gentle flux of N_2_. Samples were diluted with physiological saline up to 450–500 μL and filtered through Millex GV filters (0.22 μm, 13 mm diameter, Millipore, Milford, CT, USA). Sample radioactivity was measured in the dose calibrator (CURIEMENTOR 4, PTW Freiburg-GmbH; Freiburg, Germany) and aliquots from each sample were analyzed by radio-HPLC. Analyses were performed on a Waters Chromatograph, equipped with a 2998-photodiode array UV detector (Waters, Vienna, Austria) and a Gabi gamma detector (Raytest RSM Analytische Instrumente GmbH, Straubenhardt, Germany) with the Empower 2 Software (Waters, Milford, MA, USA) applied for data acquisition and processing. An XBridge Shield RP18 (5 μm, 4.6 mm × 20 mm) cartridge column (Waters; Vienna, Austria) was eluted at a flow rate of 1 mL/min by 0.1% TFA in H_2_O (A) and MeCN (B) with the following linear gradient system: 100%A/0%B at 0 min, with linearly increases in B by 1%/min to 60%A/40%B (system 2). The *t*_R_ of the intact radiopeptide was determined by coinjection with the respective reference in the HPLC.

#### 2.4.2. Biodistribution

The biodistribution was studied in BALB/C nu/nu mice bearing PC-3 tumors in their flanks for [^111^In]In-AU-RM26-M1 (reference; at 4 h pi), [^111^In]In-AU-RM26-M2 (at 4 h and 24 h pi), [^111^In]In-AU-RM26-M3 (at 4 h pi), and [^111^In]In-AU-RM26-M4 (at 4 h and 24 h pi); groups of four animals were used for each time point per compound. The mice were iv injected with 40 pmol peptide (30 kBq in a total volume of 100 μL 1% bovine serum albumin (BSA) in PBS). To assess in vivo GRPR specificity of the uptake of [^111^In]In-AU-RM26-M2 and [^111^In]In-AU-RM26-M4, two additional 4 h groups of 3 mice each were co-injected with an excess of NOTA-PEG2-RM26 (5 nmol, 100 μL 1% BSA in PBS) [26] together with the radioligand (30 kBq, 40 pmol). At the indicated time points, mice were euthanized under anesthesia. The organs of interest were collected, weighed, and the sample radioactivity content was measured on the gamma counter. The percentage of injected activity per gram (%IA/g) of collected tumors, organs and tissues, the %IA for the remaining gastrointestinal tract and carcass, as well as the tumor-to-organ ratios (T/O) were calculated.

#### 2.4.3. SPECT/CT Imaging

SPECT/CT imaging was performed in two different mice for [^111^In]In-AU-RM26-M2 and [^111^In]In-AU-RM26-M4 both at 4 h pi (under anesthesia) and at 24 h pi (after euthanasia with CO_2_); a bolus of the radioligand (1 MBq, 40 pmol in 100 μL of 1% BSA in PBS) was iv injected in each mouse. Whole body scans were performed using nanoScan SPECT/CT (Mediso Medical Imaging Systems, Budapest, Hungary). The acquisition time was 20 min. SPECT raw data was reconstructed using Tera-TomoTM 3D SPECT reconstruction technology (version 3.00.020.000; Mediso Medical Imaging Systems Ltd., Budapest, Hungary). CT data was reconstructed using Filter Back Projection and fused with SPECT files using Nucline 2.03 Software (Mediso Medical Imaging Systems Ltd., Budapest, Hungary).

## 3. Results

### 3.1. Radiolabeling and Radiochemical Stability

**Figure 2:**
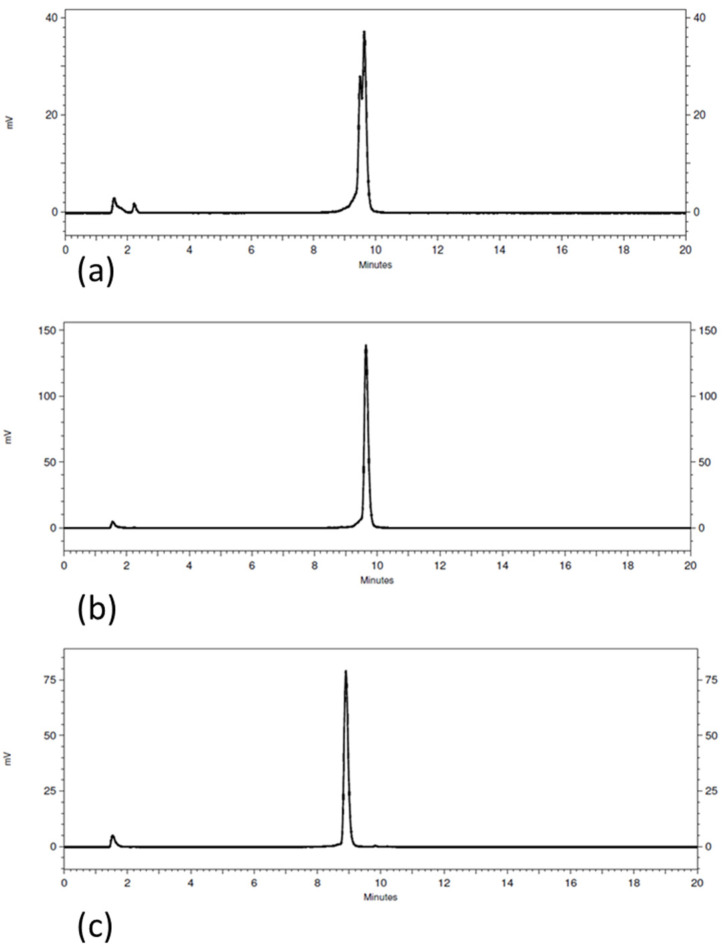
HPLC radiochromatograms of (**a**) [^111^In]In-AU-RM26-M2 (*t_R_* 9.5 min/9.6 min), (**b**) [^111^In]In-AU-RM26-M3 (*t_R_* 9.6 min), and (**c**) [^111^In]In-AU-RM26-M4 (*t_R_* 9.8 min) (system 1).

The radiochemical purity, as determined by HPLC, was around 93% for [^111^In]In-AU-RM26-M2 taking into consideration the two major peaks (retention time (*t*_R_) of 9.5 min and 9.7 min; system 1 in Section 2.2; Figure 2a). The radiochemical purity was around 96% for [^111^In]In-AU-RM26-M3 (Figure 2b) and 94% for [^111^In]In-AU-RM26-M4 (Figure 2c) and the overall radiochemical results are presented in the table below (Table 1).

### 3.2. In Vitro Studies

#### 3.2.1. In Vitro GRPR Binding Specificity

**Figure 3:**
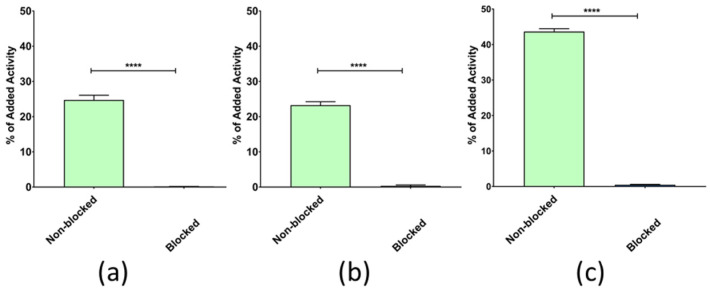
In vitro specificity assay of (**a**) [^111^In]In-AU-RM26-M2, (**b**) [^111^In]In-AU-RM26-M3, and (**c**) [^111^In]In-AU-RM26-M4 without (green bars) and in the presence of excess NOTA-PEG2-RM26 (GRPR blocking; violet bars). The error bars (not visible for some data points because they are smaller than data point symbols) represent sd, and **** corresponds to *p* < 0.0001.

The radiolabeled peptides showed a significantly lower uptake (below 0.5%) in PC-3 cells pretreated with an excess of NOTA-PEG2-RM26 for GRPR blocking than in control cells (Figure 3). This fact demonstrates the high GRPR specificity of uptake of the three new radioligands in the cells. Notably, [^111^In]In-AU-RM26-M4 displayed a 2-fold higher cell uptake in this series of analogs.

#### 3.2.2. GRPR-Affinity Measurements

**Figure 4:**
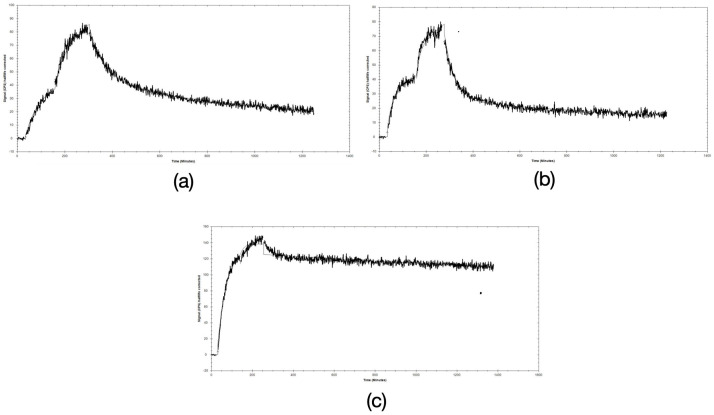
Ligand tracer sensorgrams, using a 1:2 fitting interaction model, of (**a**) [^111^In]In-AU-RM26-M2, (**b**) [^111^In]In-AU-RM26-M3, and (**c**) [^111^In]In-AU-RM26-M4. The association was measured at 1 and 3 nM.

The binding affinity of the [^111^In]In-labeled peptides to the human GRPR was measured in real time on PC-3 cells. The sensorgrams were analyzed with a 1:2 fitting interaction model (Figure 4). The radiolabeled variants [^111^In]In-AU-RM26-M2 and [^111^In]In-AU-RM26-M3 had similar K_D_1 and K_D_2. On the other hand, the K_D_1 and K_D_2 of [^111^In]In-AU-RM26-M4 were two orders of magnitude lower compared to [^111^In]In-AU-RM26-M2 and [^111^In]In-AU-RM26-M3 (Table 2).

#### 3.2.3. Cellular Internalization

**Figure 5:**
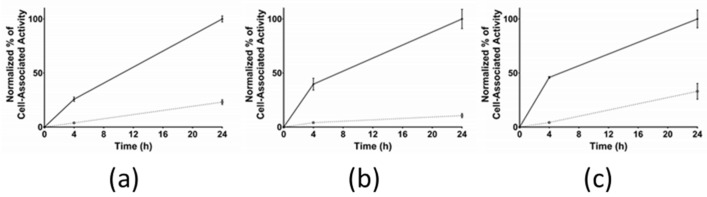
Cellular internalization of (**a**) [^111^In]In-AU-RM26-M2, (**b**) [^111^In]In-AU-RM26-M3, and (**c**) [^111^In]In-AU-RM26-M4 in PC-3 cells at 4 and 24 h; data represent the mean value from 3 dishes normalized to the maximum cell association at 24 h and the error bars indicate sd. Solid line: total cell-associated activity; dashed line: internalized fraction.

The cellular internalization for [^111^In]In-AU-RM26-M2, [^111^In]In-AU-RM26-M3, and [^111^In]In-AU-RM26-M4 was studied in GRPR-positive PC-3 cells (Figure 5). The normalized cell association activity increased over time for the three radiopeptides. Interestingly, the internalized fraction was found to be similar at 4 h, but significantly differed across analogs at 24 h according to the following rank: [^111^In]In-AU-RM26-M3 (11 ± 2%) < [^111^In]In-AU-RM26-M2 (23 ± 2%; *p* < 0.01 vs. [^111^In]In-AU-RM26-M3) < [^111^In]In-AU-RM26-M4 (33 ± 7%; *p* < 0.0001 vs. [^111^In]In-AU-RM26-M3 and *p* < 0.01 vs. [^111^In]In-AU-RM26-M2).

### 3.3. In Vivo Studies

#### 3.3.1. In Vivo Metabolic Stability

The metabolic stability of the new analogs was compared in peripheral mice blood at 5 min pi vs. the [^111^In]In-AU-RM26-M1 reference without or during NEP-inhibition (Table 3). While [^111^In]In-AU-RM26-M2 and [^111^In]In-AU-RM26-M4 were found to be comparably stable with the [^111^In]In-AU-RM26-M1 reference in control mice, [^111^In]In-AU-RM26-M3 degraded faster (*p* < 0.0001). Treatment of animals with Entresto^®^, a registered antihypertensive drug in vivo releasing the potent and selective NEP-inhibitor sacubitrilat after oral administration, resulted in significant metabolic stability improvements for [^111^In]In-AU-RM26-M2 and [^111^In]In-AU-RM26-M3, implicating NEP as the major degrading protease. For [^111^In]In-AU-RM26-M3 in particular, a 3-fold higher amount of intact radioligand was detected in the blood of the Entresto^®^ group of mice compared with controls (*p* < 0.0001).

#### 3.3.2. Biodistribution

**Figure 6:**
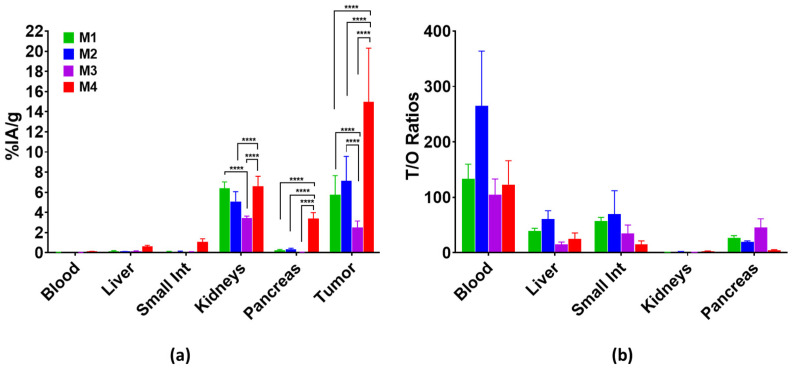
Comparative biodistribution data in BALB/C nu/nu mice bearing PC-3 xenografts selectively for blood, liver, small intestine, kidneys and tumors at 4 h pi of [^111^In]In-AU-RM26-M1 (reference; green bars), [^111^In]In-AU-RM26-M2 (blue bars), [^111^In]In-AU-RM26-M3 (violet bars), and [^111^In]In-AU-RM26-M4 (red bars); results are expressed as (**a**) average IA/g ± sd, n = 4 and (**b**) T/O ratios; statistically significant differences of *p* < 0.0001 are denoted by ****, according to a two-way Anova with Tuckey’s post hoc analysis.

The biodistribution of [^111^In]In-AU-RM26-M2, [^111^In]In-AU-RM26-M3 and [^111^In]In-AU-RM26-M4 in PC-3 xenografted BALB/C nu/nu mice was compared at 4 h pi with [^111^In]In-AU-RM26-M1 (reference); for [^111^In]In-AU-RM26-M2 and [^111^In]In-AU-RM26-M4, additional animal groups at 24 pi and during in vivo GRPR blockade at 4 h pi (by coinjection of excess NOTA-PEG2-RM26) were investigated. The results as average percentages of injected activity per gram tissue (%IA/g) ± sd (except for carcass and intestines reported as %IA ± sd) are included in Table S1, whereas the respective tumor-to-organ (T/O) ratios are presented in Table S2 (Supplementary File). For easy comparison purposes, results for PC-3 tumors, blood, excretory (intestines and kidneys) and GRPR-positive organs (intestines and pancreas) are selectively shown in Figure 6 along with the respective T/O ratios. It is interesting to observe that [^111^In]In-AU-RM26-M4 outperformed the rest of the radioligands with regards to tumor uptake (15 ± 5%IA/g > 7 ± 2%IA/g; *p* < 0.0001 ([^111^In]In-AU-RM26-M2) > 6 ± 2%IA/g; *p* < 0.0001 ([^111^In]In-AU-RM26-M1) >> 2.5 ± 0.6%IA/g; *p* < 0.0001 ([^111^In]In-AU-RM26-M3). This result is in line with the combination of the higher GRPR affinity, PC-3 cell uptake and excellent in vivo stability of the radioligand. All analogs showed low radioactivity levels in the blood (<0.15%IA/g), gastrointestinal tract (GIT; not emptied of its contents < 2%IA) and the whole body (<5%IA). [^111^In]In-AU-RM26-M4 showed higher uptake in the GRPR-positive organs, especially in mouse pancreas (3.4 ± 0.6 %IA/g > 0.37 ± 0.08%IA/g; *p* < 0.0001 ([^111^In]In-AU-RM26-M2). Likewise, higher radioactivity levels were observed for [^111^In]In-AU-RM26-M4 in most physiological tissues. The blood and liver radioactivity values were very comparable for [^111^In]In-AU-RM26-M1, [^111^In]In-AU-RM26-M2 and [^111^In]In-AU-RM26-M3, whereas [^111^In]In-AU-RM26-M4 displayed higher, but not significantly higher, levels in the blood (0.125 ± 0.0004%IA/g; *p* > 0.05). The low liver and GIT radioactivity indicated excretion predominantly via the kidneys for all analogs. [^111^In]In-AU-RM26-M3 had the lowest kidney uptake (~3.5%IA/g) that was significantly lower than for [^111^In]In-AU-RM26-M1 (~6.5%IA/g; *p* < 0.0001) and [^111^In]In-AU-RM26-M4 (7.2 ± 0.7%IA/g; *p* < 0.0001). As a result, [^111^In]In-AU-RM26-M4 displayed less advantageous T/O ratios in most physiological organs compared with [^111^In]In-AU-RM26-M2, which exhibited overall a favorably clearer background at 4 h pi. On the other hand, the tumor-to-kidney ratio turned out to be in favor of [^111^In]In-AU-RM26-M4 (2.3 vs. 1.6 for [^111^In]In-AU-RM26-M2).

**Figure 7:**
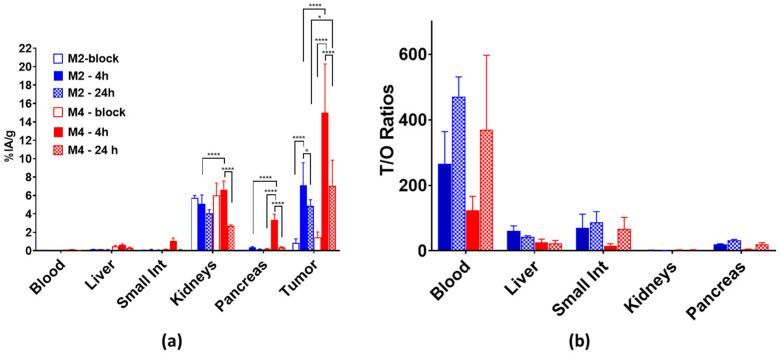
Comparative biodistribution data in BALB/C nu/nu mice bearing PC-3 xenografts selectively for blood, liver, small intestine, kidneys and tumors at 4 h block (empty-fill bars), 4 h (solid-fill bars), and 24 pi (chequered-fill bars) of [^111^In]In-AU-RM26-M2 (blue bars) and [^111^In]In-AU-RM26-M4 (red bars); results are expressed as (**a**) average IA/g ± sd, n = 4 and (**b**) T/O ratios; statistically significant differences of *p* < 0.0001 are denoted by **** and *p* < 0.05 by *, according to a two-way Anova with Tuckey’s post hoc analysis.

We next present results assessing the impact of GRPR-mediated contribution in the uptake of [^111^In]In-AU-RM26-M2 and [^111^In]In-AU-RM26-M4 in the tumors and in physiological organs, as well as comparing the radioactivity washout from the tumors and the background between these two best-performing radioligands. This additional data from animal groups during in vivo GRPR blockade at 4 h pi and at 24 h pi is presented in Figure 7. The tumor uptake of both radioligands was found to be GRPR-specific at 4 h pi by the significantly reduced uptake seen in the block-groups of animals ([^111^In]In-AU-RM26-M2: 7 ± 2%IA/g in controls vs. 0.9 ± 0.4%IA/g in blocks; *p* < 0.0001 and [^111^In]In-AU-RM26-M4: 15 ± 5%IA/g in controls vs. 1.4 ± 0.6%IA/g in blocks; *p* < 0.0001) and a similar trend was also evident in the GRPR-rich mouse pancreas. Significant washout of radioactivity was observed between 4 and 24 h pi from the implanted tumor, for both radioligands, with [^111^In]In-AU-RM26-M4 preserving less than half of its 4 h value, but still showing a higher uptake over [^111^In]In-AU-RM26-M2 at 24 h pi (7 ± 3%IA/g and 4.9 ± 0.7%IA/g (70% of the 4 h value), respectively; *p* < 0.05). On the other hand, the washout of [^111^In]In-AU-RM26-M4 from physiological tissues was much faster. For example, in the pancreas, it displayed less than 10% of the initial 4 h uptake, whereas the respective 24 h value of [^111^In]In-AU-RM26-M2 represented 40% of the 4 h uptake. A similar trend was observed in most background organs, including the kidneys with [^111^In]In-AU-RM26-M4 at 24 h pi retaining < 38% and [^111^In]In-AU-RM26-M2 > 80% of their 4 h renal values. It should be noted that the background washout patterns of both radioligands are characteristic for GRPR-antagonists. However, [^111^In]In-AU-RM26-M4 displayed increases in the T/O ratios between 4 and 24 h pi whereby such ratios changed less advantageously in the case of [^111^In]In-AU-RM26-M2 in most organs.

#### 3.3.3. SPECT/CT Imaging

**Figure 8:**
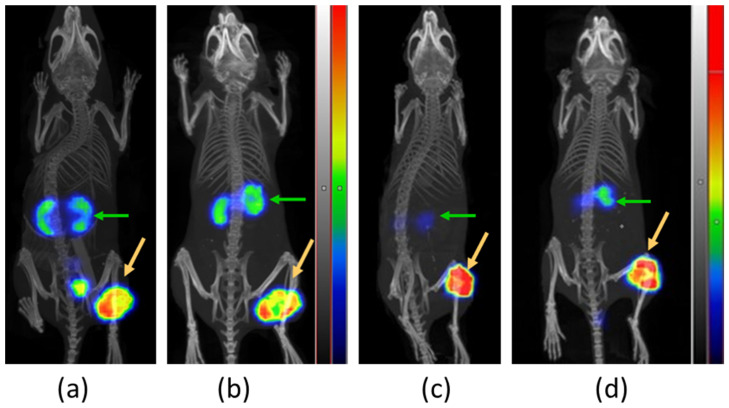
Comparative SPECT/CT images of mice bearing PC-3 xenografts for [^111^In]In-AU-RM26-M2 at (**a**) 4 h and (**b**) 24 h pi and for [^111^In]In-AU-RM26-M4 at (**c**) 4 h and (**d**) 24 h pi; green arrows are directed toward the kidneys and orange arrows at the implanted PC-3 tumors; scans were presented as maximum intensity projections in the red/green/blue color scale.

Comparative SPECT/CT images were acquired from two BALB/C nu/nu mice bearing PC-3 tumors in their flanks at 4 and 24 h pi of [^111^In]In-AU-RM26-M2 (first mouse) and [^111^In]In-AU-RM26-M4 (second mouse) and are presented in Figure 8. Concordant with biodistribution results, the PC-3 tumors and the kidneys were visualized at both time points against a clear background. [^111^In]In-AU-RM26-M4 displayed higher tumor uptake and lower renal radioactivity compared with [^111^In]In-AU-RM26-M2 at both time intervals.

## 4. Discussion

The development of new peptide radiopharmaceuticals for cancer theranostics is a tedious and iterative process. Structural modifications, no matter how small, may significantly impact critical biological features, such as receptor affinity, uptake/internalization in tumor cells, stability in the biological milieu (both with regards to radiometal-chelate integrity and to cleavage of peptide bonds by endogenous peptidases) and eventually biodistribution patterns [35]. During the developmental process, the existing body of literature data becomes essential for selecting the most promising modifications to implement. Accordingly, the new GRPR radioligands in the present study are in fact Gly^11^/Sar^11^-substituted mimics of [^111^In]In-DOTAGA-PEG2-RM26, based on previous observations that related Gly^11^/Sar^11^-modified analogs, such as [^99m^Tc]Tc-DB15 [18] or [^111^In]In-AU-RM26-M1 [26], exhibit higher in vivo stability without impairment of other biological properties. Furthermore, enhancement of receptor affinity and cell uptake were reported for peptide radioligands with positively charged residues at the N-terminal region [7,19,27,28]. This finding was also exploited in the design of the new [^111^In]In-AU-RM26-M2/3/4 analogs. As shown in Figure 1, the type and number of basic residues in the linker varied to include either single (Pip or Arg) or multiple (Arg-Arg-Pip) basic sites. Still, amino acids with lateral primary amines (e.g., Lys, Dab) were excluded from this search, due to their propensity to unfavorably enhance kidney uptake [7,19,29].

The prospects of AU-RM26-M2/3/4, seen together as theranostic In-111/Lu-177 pair candidates (for SPECT diagnostic imaging/radionuclide therapy), were first assessed in the In-111 analogs and in comparison with the [^111^In]In-AU-RM26-M1 reference [26]. In this way, the best performing compound(s) could be selected for future evaluation in combination with the respective [^177^Lu]Lu-labeled therapeutic counterpart(s). Unsurprisingly, incorporation of In-111 by DOTAGA was successful for all new peptide conjugates, resulting in high-quality and high-purity radioligands. The [^111^In]In-DOTAGA radiometal-chelate was found to be stable both in human serum and during challenge conditions. In view of the above, all subsequent biological experiments proceeded without further purification of radiolabeled products (Table 1, Figure 2).

The new radioligands were efficiently taken up by PC-3 cells via a GRPR-driven process, as established by the drastic drop of uptake observed in the presence of excess GRPR blockers (Figure 3). Interestingly, [^111^In]In-AU-RM26-M4, containing the Arg-Arg-Pip triad of basic amino acids, achieved twice as high uptake in the cells compared with the other two analogs, containing each a single basic residue in the linker. This finding revealed the favorable impact of positive charges in the linker on cell uptake. Concordant with this result, [^111^In]In-AU-RM26-M4 displayed higher affinity for the GRPR with both K_D_1 and K_D_2 being more than two orders of magnitude lower than for either [^111^In]In-AU-RM26-M2 or [^111^In]In-AU-RM26-M3 (Figure 4, Table 2) or previously tested [^111^In]In-AU-RM26-M1 [26]. This result further supports the beneficial presence of the Arg-Arg-Pip basic triplet in the N-terminal region of [^111^In]In-AU-RM26-M4 on receptor affinity, confirming our original hypothesis. In a last set of experiments, the cellular internalization of the three radiolabeled GRPR-antagonists was tested in a period of up to 24 h (Figure 5). All three [^111^In]In-AU-RM26-M2/3/4 displayed a typical GRPR-antagonist profile with the bulk of cell-associated radioactivity bound on the cell membrane, especially in the early time point of 4 h [18]. The internalization pattern of the new radiopeptides was similar to the previously studied antagonist [^111^In]In-AU-RM26-M1 [26]. Radiolabeled antagonists do not typically internalize in target cells, although radioactivity distribution patterns tend to change in the cells over time. This process is most probably related to the constitutive internalization of the receptor/receptor–radioligand complex, with the radioactivity shifting little by little from the cell membrane into cells [18]. Indeed, we observe slowly increasing intracellular levels of radioactivity for all analogs between 4 and 24 h. Internalization levels become clearly distinct across radioligands at 24 h pi, with [^111^In]In-AU-RM26-M4 shifting significantly faster from the cell membrane into cells compared to the other two analogs (Figure 5). This result is of great interest in view of the therapeutic prospects of these analogs, when seen as an indicator of radioactivity retention times in tumor cells in vivo, a crucial element of therapeutic efficacy [25].

Peptide-based drugs, and consequently peptide radiopharmaceutical candidates as well, need to overcome their major inherent drawback, namely rapid degradation in the biological milieu. Omnipotent peptidases, cleaving one or more peptide bonds in the peptide chain, often compromise their efficacy and clinical applicability options [36,37,38]. Notably, peptide-based radiopharmaceuticals are almost exclusively iv injected to patients, becoming immediately exposed to peptidases in the blood solute and those anchored on epithelial cells of vasculature and major tissues of the body, like NEP [39,40,41]. Luckily, most peptide radioligands are N-capped via the attachment of the radiometal-chelate, becoming stable against N-exopeptidases [39]. In general, radiopeptides reach their tumor-targets quite fast, but the detrimental action of peptidases is actually even faster. To counterbalance this handicap, the new ligands presented herein are Gly^11^/Sar^11^-substituted, a modification previously shown to enhance resistance to NEP [18,26]. However, the introduction of basic residues in the linker induced a few unexpected results (Table 3). While [^111^In]In-AU-RM26-M1 (reference) and [^111^In]In-AU-RM26-M4 (Arg-Arg-Pip modified) showed similarly high stability in mice blood circulation, [^111^In]In-AU-RM26-M2 (Pip modified) showed a slight drop in stability and [^111^In]In-AU-RM26-M3 (Arg modified) was drastically less stable in mice circulation. In all cases, the involvement of NEP could be confirmed via the significant stability improvements during NEP inhibition. The latter was induced in mice following per os treatment with Entresto^®^, as a source of the potent and selective NEP-inhibitor sacubitrilat [31,32,33,34]. Improvements were more clearly visible for the less stable analogs, especially [^111^In]In-AU-RM26-M3 (Table 3). Apparently, Arg in direct vicinity to the N-terminal DPhe^6^ in [^111^In]In-AU-RM26-M3 turned out to negatively affect stability, in contrast to Pip ([^111^In]In-AU-RM26-M2) and Arg-Arg-Pip ([^111^In]In-AU-RM26-M4). This finding demonstrates that both the number and the type of positive charges affect peptides’ stability. We might speculate that such charge variations lead to different orientation and docking of the whole molecule to the active site of the degrading enzyme.

The biodistribution patterns of the three new radioligands and the reference were directly compared in mice bearing PC-3 xenografts at 4 h pi as a first screening to establish which structural modifications have been advantageous in this set of analogs. Taking tumor uptake as a major criterion, we observe that [^111^In]In-AU-RM26-M4 outperformed any other candidate (Figure 6; Table S1). This result can be attributed to a combination of qualities synergistically operating, including the high in vivo stability, superior affinity for GRPR and significantly higher uptake and intracellular residence times in PC-3 cells in vitro. On the other end of tumor uptake, [^111^In]In-AU-RM26-M3 combines a quite poor in vivo stability with less affinity and uptake in the cells. Interestingly, the positive charge of Arg adjacent to N-terminal DPhe^6^ failed to enhance receptor affinity, while being quite disadvantageous with regards to stability against NEP. We see that subtle changes at this region of the radiopeptide have a tremendous effect on stability, as previously reported [18]. It further revealed that Gly^11^/Sar^11^-substitution alone does not suffice for full resistance to this omnipresent peptidase. Replacing Arg by Pip in [^111^In]In-AU-RM26-M2 changed this scenario again. In this case, we note very good resistance to NEP in combination with a good receptor affinity and PC-3 cell uptake, leading to second-best tumor values for [^111^In]In-AU-RM26-M2. The biodistribution of the reference peptide [^111^In]In-AU-RM26-M1 was similar to [^111^In]In-AU-RM26-M2, but with somewhat higher activity uptake in kidneys and lower uptake in tumors.

For high-quality imaging as well as a favorable therapeutic index, tumor uptake is not the sole criterion, but needs to be evaluated together with a fast background clearance. Tumor uptake and a rapidly declining background are taken together into serious consideration during screening of new analogs, especially when therapeutic applications are envisaged as a next step of development. Both these parameters affect radiotoxicity, therapeutic efficacy, dosimetric calculations and are essential for therapy planning. Consequently, T/O ratios represent another important criterion. In this respect, [^111^In]In-AU-RM26-M2 showed better results compared with [^111^In]In-AU-RM26-M4, with the exception of the kidneys (Figure 6; Table S2). The GRPR expression in physiological tissues, such as the mouse pancreas and intestinal tube, is expected to affect radioactivity levels of the two analogs taken together with the higher stability and receptor affinity of [^111^In]In-AU-RM26-M4. On the other hand, antagonists are known to clear faster from physiological tissues than tumors [18].

To better evaluate the two best-performing analogs considering these parameters, a head-to-head comparison was performed both at 4 h pi during GRPR blockade and at 24 h (Figure 7; Tables S1 and S2). Indeed, the uptake of [^111^In]In-AU-RM26-M4 in various GRPR-rich mice tissues could be attributed to its higher receptor affinity compared to [^111^In]In-AU-RM26-M2. On the other hand, [^111^In]In-AU-RM26-M42 uptake declined more promptly between 4 and 24 h in most tissues compared to [^111^In]In-AU-RM26-M2 and hence the T/O-ratios increased at a greater rate for the Arg-Arg-Pip carrying radioligands.

This result was highly evident in the SPECT/CT images (Figure 8). Several factors need to be evaluated before adopting this conclusion from In-111 to Lu-177 and from mice to humans. First, the coordination chemistry of In-111 and Lu-177 to DOTAGA may lead to subtle physicochemical differences between the radiotheranostic pair members with effects on the biodistribution patterns. Second, tumor and background levels could be “tuned” to a certain extent by adjustments of peptide doses and molar activity. Finally, translation from mice models to patients may result in further differing patterns.

## 5. Conclusions

We herein compared three [^111^In]In-AU-RM26-M1 mimics carrying the [^111^In]In-DOTAGA radiometal-chelate at the N-terminal DPhe^6^ of [Sar^11^]RM26 through positively charged linkers. While the Gly^11^/Sar^11^ substitution was pursued for increasing in vivo stability, the introduction of basic residues in the linker aimed to improve receptor affinity and enhance the uptake in GRPR-expressing cells and tumor models in mice. Both the type and the number of basic residues in the linker were shown to exert a profound impact on a series of important biological features of the new analogs, such as receptor affinity, uptake and internalization in PC-3 cells, in vivo stability and biodistribution patterns in mice models. [^111^In]In-AU-RM26-M4 with an Arg-Arg-Pip triplet as a linker displayed the highest tumor uptake, while requiring longer times to clear from the background. On the other hand, [^111^In]In-AU-RM26-M2 (PEG2-Pip linker) showed faster background clearance, albeit lower tumor uptake in mice. The validity of these results should be next confirmed for the Lu-177 therapeutic counterparts to establish the applicability prospects of the In-111/Lu-177 analogs as radiotheranostic pair candidates in human prostate cancer.

## Figures and Tables

**Table 1:** Radiochemical results for [^111^In]In-AU-RM26-M2, [^111^In]In-AU-RM26-M3 and [^111^In]In-AU-RM26-M4 shown as average percentage values ± standard deviation (sd) with repetitions given in parentheses. Results for incubation with PBS and in presence of EDTA are given as the percentages of released [^111^In]In^3+^.

Compound	iTLC * RCY (%) (n)	×1000 EDTA (1 h) (n)	PBS (n)	HPLC ** RCP (%)
[^111^In]In-AU-RM26-M2	97 ± 3 (7)	5.7 ± 0.7% (3)	5.7 ± 0.2% (3)	93 ± 3
[^111^In]In-AU-RM26-M3	98 ± 2 (5)	3.9 ± 0.3% (3)	3.7 ± 0.7% (3)	96.1 ± 0.8
[^111^In]In-AU-RM26-M4	99 ± 1 (5)	3.2 ± 0.1% (3)	1.9 ± 0.1% (3)	94 ± 2

* iTLC conditions and ** HPLC system 1 are detailed in Section 2.2.

**Table 2:** Affinity measurements of [^111^In]In-AU-RM26-M2, [^111^In]In-AU-RM26-M3 and [^111^In]In-AU-RM26-M4.

Interaction Constants	[^111^In]In-AU-RM26-M2	[^111^In]In-AU-RM26-M3	[^111^In]In-AU-RM26-M4
k_a_1 (M^−1^s^−1^)	7.93 × 10^4^	1.02 × 10^5^	3.44 × 10^5^
k_d_1 (s^−1^)	2.50 × 10^−5^	2.50 × 10^−5^	2.00 × 10^−6^
K_D_1 (M)	3.15 × 10^−10^	2.45 × 10^−10^	5.80 × 10^−12^
k_a_2 (M^−1^s^−1^)	1.39 × 10^5^	2.82 × 10^5^	3.46 × 10^5^
k_d_2 (s^−1^)	2.33 × 10^4^	3.94 × 10^−4^	2.08 × 10^−6^
K_D_2 (M)	1.68 × 10^−9^	1.40 × 10^−9^	6.0 × 10^−12^

Note: k_a_ = association constant, k_d_ = dissociation constant, K_D_ = equilibrium dissociation constant.

**Table 3:** Metabolic stability of [^111^In]In-DOTAGA-PEG2-RM26, [^111^In]In-AU-RM26-M1, [^111^In]In-AU-RM26-M2, [^111^In]In-AU-RM26-M3 and [^111^In]In-AU-RM26-M4 in peripheral mice blood at 5 min pi; control: mice iv injected only with the radioligand (n = 3), Entresto^®^: animals treated per os with Entresto^®^ 30 min prior to radioligand injection (n = 3). Results represent % intact radioligand detected in mice blood ± sd.

Compound	Control	Entresto^®^ Treated	Control/ Entresto^®^
[^111^In]In-AU-RM26-M1 (ref.)	88 ± 8	82 ± 1 ^NS^	^NS^
[^111^In]In-AU-RM26-M2	78 ± 2 *	91 ± 2 ^NS^	*p* < 0.001
[^111^In]In-AU-RM26-M3	25 ± 5 ***	94 ± 0.8 ^NS^	*p* < 0.0001
[^111^In]In-AU-RM26-M4	83 ± 2 ^NS^	92 ± 1 ^NS^	*p* < 0.05

^NS^: non-significant; ref: reference; *: *p* < 0.05 vs. reference; ***: *p* < 0.0001 vs. reference.

## Data Availability

Data is contained within the article and in the Supplementary File.

## Supplementary Materials

The following supporting information can be downloaded at: https://www.mdpi.com/article/10.3390/pharmaceutics16040513/s1, Table S1: Biodistribution data (%IA/g ± sd, n = 4) of [^111^In]In-AU-RM26-M1 (4 h pi), [^111^In]In-AU-RM26-M2 (4 h pi controls and GRPR-blocked; 24 h pi), [^111^In]In-AU-RM26-M3 (4 h pi), and [^111^In]In-AU-RM26-M4 (4 h pi controls and GRPR-blocked; 24 h pi) in PC-3 xenografted mice, Table S2: Tumor-to-organ ratios of [^111^In]In-AU-RM26-M1 (4 h pi), [^111^In]In-AU-RM26-M2 (4 and 24 h pi), [^111^In]In-AU-RM26-M3 (4 h pi) and [^111^In]In-AU-RM26-M4 (4 and 24 h pi) in PC-3 tumor-bearing mice.
