# Supplementary material for: GRPR-Antagonists Carrying DOTAGA-Chelator via Positively Charged Linkers: Perspectives for Prostate Cancer Theranostics
Source: Pharmaceutics. 2024 Apr 8;16(4):513. doi: 10.3390/pharmaceutics16040513 (PMC11054746; doi:10.3390/pharmaceutics16040513)
Supplement: Supplementary file 1 [file pharmaceutics-16-00513-s001.zip › pharmaceutics-2944626-supplementary.pdf]

Supplementary data

GRPR-antagonists carrying DOTAGA-chelator via positively-charged linkers: Perspectives for prostate cancer theranostics

Karim Obeid , Panagiotis Kanellopoulos, Ayman Abouzayed, Adam Mattsson, Vladimir Tolmachev, Berthold A. Nock, Theodosia Maina , Anna Orlova

Table S1. Biodistribution data (%IA/g ± sd, n= 4) of [^{111}In]In-AU-RM26-M1, [^{111}In]In-AU-RM26-M2, [^{111}In]In-AU-RM26-M3, and [^{111}In]In-AU-RM26-M4 in PC-3 xenografted mice; at 4 h pi for [^{111}In]In-AU-RM26-M1 and [^{111}In]In-AU-RM26-M3 and at 4 h and 24 h pi for [^{111}In]In-AU-RM26-M2 and [^{111}In]In-AU-RM26-M4; results during in vivo GRPR-blockade by coinjection of excess NOTA-PEG2-RM26 at 4 h pi for [^{111}In]In-AU-RM26-M2 and [^{111}In]In-AU-RM26-M4 are also included (Block, values shown in italics; n= 3). Uptake in GIT and carcass is given as %IA ± sd (n= 4).

Organ	M1	M2		M3	M4	
	4 h	4 h	24 h	4 h	4 h	24 h
**Blood**	0.04 ± 0.01	0.003 ± 0.002<br>*0.06 ± 0.01*	0.01 ± 0.002	0.024 ± 0.002	0.122 ± 0.0013<br>*0.08 ± 0.01*	0.02 ± 0.01
**Lungs**	0.08 ± 0.05	0.05 ± 0.01<br>*0.07 ± 0.01*	0.05 ± 0.02	0.12 ± 0.01	0.2 ± 0.08<br>*0.10 ± 0.0002*	0.06 ± 0.02
**Liver**	0.2 ± 0.1	0.12 ± 0.01<br>*0.15 ± 0.01*	0.11 ± 0.01	0.17 ± 0.01	0.6 ± 0.11<br>*0.45 ± 0.11*	0.31 ± 0.07
**Spleen**	0.08 ± 0.02	0.07 ± 0.02<br>*0.07 ± 0.01*	0.07 ± 0.01	0.08 ± 0.01	0.23 ± 0.07<br>*0.12 ± 0.004*	0.14 ± 0.04
**Pancreas**	0.2 ± 0.1	0.37 ± 0.08[e]<br>*0.05 ± 0.01*	0.15 ± 0.03	0.06 ± 0.01[f]	3.4 ± 0.6[e,f,h]<br>*0.20 ± 0.05*[j]	0.36 ± 0.08[h,j]
**Small Int**	0.10 ± 0.04	0.10 ± 0.06<br>*0.06 ± 0.02*	0.06 ± 0.03	0.08 ± 0.03	1.1 ± 0.3<br>*0.13 ± 0.04*	0.11 ± 0.03
**Kidneys**	6.4 ± 0.6[b,g]	5 ± 1<br>*5.7 ± 0.3*	4.1 ± 0.4	3.4 ± 0.2[b,f]	7 ± 1[g,f,h]<br>*6 ± 1*	2.7 ± 0.1[h]
**Tumor**	6 ± 2[b,c]	7 ± 2[d,e,g,j]<br>*0.9 ± 0.4*[j]	4.9 ± 0.7[g,k]	2.5 ± 0.6[b.d,f]	15 ± 5[c,e,f,h,j]<br>*1.4 ± 0.6*[j]	7 ± 3[h,k]
**Muscle**	0.04 ± 0.02	0.02 ± 0.01<br>*0.02 ± 0.003*	0.02 ± 0.01	0.03 ± 0.01	0.06 ± 0.02<br>*0.05 ± 0.02*	0.02 ± 0.01
**Bone**	0.05 ± 0.01	0.05 ± 0.03<br>*0.05 ± 0.01*	0.04 ± 0.02	0.06 ± 0.01	0.13 ± 0.04<br>*0.08 ± 0.004*	0.09 ± 0.02
**GI**	0.9 ± 0.1	0.8 ± 0.5<br>*0.46 ± 0.03*	0.13 ± 0.03	1.3 ± 0.9	1.4 ± 0.3<br>*0.5 ± 0.2*	0.15 ± 0.03
**Carcass**	1.3 ± 0.5	0.8 ± 0.1[e]<br>*1 ± 0.5*	0.5 ± 0.2	1.7 ± 0.3	3 ± 1.5[e,h]<br>*1.6 ± 0.3*	0.8 ± 0.2[h]

[a-k] Two-way Anova with Tuckey's post hoc analysis ($p < 0.05$): [a] Significant difference between [^{111}In]In-AU-RM26-M1 and [^{111}In]In-AU-RM26-M2; [b] Significant difference between [^{111}In]In-AU-RM26-M1 and [^{111}In]In-AU-RM26-M3; [c] Significant difference between [^{111}In]In-AU-RM26-M1 and [^{111}In]In-AU-RM26-M4; [d] Significant difference between [^{111}In]In-AU-RM26-M2 and [^{111}In]In-AU-RM26-M3; [e] Significant difference between [^{111}In]In-AU-RM26-M2 and [^{111}In]In-AU-RM26-M4 at 4 h pi; [f] Significant difference between [^{111}In]In-AU-RM26-M3 and [^{111}In]In-AU-RM26-M4; [g] Significant difference between [^{111}In]In-AU-RM26-M2 at 4 and 24 h pi; [h] Significant difference between [^{111}In]In-AU-RM26-M4 at 4 and 24 h pi; [i] Significant difference between [^{111}In]In-AU-RM26-M2 4 h and block; [j] Significant difference between [^{111}In]In-AU-RM26-M4 4 h and block; [k] Significant difference between [^{111}In]In-AU-RM26-M3 and [^{111}In]In-AU-RM26-M4 at 24 h pi; unless clearly stated, differences refer to values at 4 h pi.

Table S2. Tumor-to-organ ratios of [^{111}In]In-AU-RM26-M1 (M1), [^{111}In]In-AU-RM26-M2 (M2), [^{111}In]In-AU-RM26-M3 (M3) and [^{111}In]In-AU-RM26-M4 (M4) in PC-3 tumor-bearing mice; for [^{111}In]In-AU-RM26-M1 and [^{111}In]In-AU-RM26-M3 at 4 h pi and for [^{111}In]In-AU-RM26-M2 and [^{111}In]In-AU-RM26-M4 at 4 h and 24 h pi.

Organ	M1	M2		M3	M4	
	4 h	4 h	24 h	4 h	4 h	24 h
**Blood**	133 ± 26	265 ± 99^{c,d,e}	471 ± 60^{c}	104^{d} ± 29	123 ± 43^{e,g}	369 ± 228^{g}
**Lungs**	81 ± 25	173 ± 50	101 ± 37	33 ± 22	90 ± 59	120 ± 61
**Liver**	39 ± 5	60 ± 16	43 ± 3	15 ± 4.5	25 ± 11	23 ± 9
**Spleen**	72 ± 9.5	109 ± 15	74 ± 20	31 ± 9	71 ± 37	54 ± 30
**Pancreas**	27 ± 4	19 ± 2	32 ± 3	45 ± 15	4 ± 1	20 ± 5
**Small Int**	57 ± 7	70 ± 42	87 ± 33	35 ± 15	15 ± 7	67 ± 35
**Kidneys**	0.9 ± 0.2	1.6 ± 0.7	1.20 ± 0.15	0.7 ± 0.2	2.3 ± 0.9	2.6 ± 0.9
**Muscle**	234 ± 154^{a,b}	478 ± 298^{a,c,d,e}	313 ± 127^{c}	**75** ± 18^{b,d,f}	307 ± 168^{e,f}	347 ± 176
**Bone**	117 ± 16	184 ± 156	157 ± 91	39 ± 8	119 ± 40	79 ± 25

Two-way Anova with Tuckey’s post hoc analysis ($p < 0.05$): ^{a} Significant difference between [^{111}In]In-AU-RM26-M1 and [^{111}In]In-AU-RM26-M2 (4 h pi); ^{b} Significant difference between [^{111}In]In-AU-RM26-M1 and [^{111}In]In-AU-RM26-M3 (4 h pi); ^{c} Significant difference between [^{111}In]In-AU-RM26-M2 4 h and 24 h pi; ^{d} Significant difference between [^{111}In]In-AU-RM26-M2 (4 h pi) and [^{111}In]In-AU-RM26-M3 (4 h pi); ^{e} Significant difference between [^{111}In]In-AU-RM26-M2 (4 h pi) and [^{111}In]In-AU-RM26-M4 (4 h pi); ^{f} Significant difference between [^{111}In]In-AU-RM26-M3 and [^{111}In]In-AU-RM26-M4 (4 h pi); ^{g} Significant difference between [^{111}In]In-AU-RM26-M4 4 h pi and 24 h pi.
